# Characterization of spontaneously-developed non-alcoholic fatty liver disease in aged rhesus monkeys

**DOI:** 10.1186/s13098-018-0370-1

**Published:** 2018-09-10

**Authors:** Wen Zheng, Yuli Liu, Haibao Shang, Yan Zhang, Dongwei Ma, Ning Hou, Jue Wang, Xueting Sun, Ying Peng, Lin Pan, Zhilong Wang, Xinran Tang, Rui-Ping Xiao, Xiuqin Zhang

**Affiliations:** 1 Institute of Molecular Medicine, Peking University, Beijing, 100871 China; 2 Beijing Key Laboratory of Cardiometabolic Molecular Medicine, Peking University, Beijing, 100871 China; 3 Laboratory Animal Center, Peking University, Beijing, 100871 China; 4 Peking University Third Hospital, Beijing, 100191 China; 5 China-Japan Friendship Hospital, Beijing, 100029 China; 6 Peking University People’s Hospital, Beijing, 100044 China; 7 INC Research, Beijing, 100020 China; 8 Peking-Tsinghua Center for Life Sciences, Peking University, Beijing, China

**Keywords:** Non-alcoholic fatty liver disease, Non-human primates, Ultrasonographic steatosis score, Metabolic syndrome

## Abstract

**Background:**

Non-alcoholic fatty liver disease (NAFLD) is a global epidemic afflicting 20–30% in the general population. The animal model of NAFLD available at the present are less clinically relevant. In this study. We aimed to establish a NAFLD model of rhesus monkeys and develop an ultrasonographic steatosis score (USS) system to grade hepatic steatosis in this model.

**Methods:**

We performed hepatic ultrasonography and blood biochemical tests on 86 rhesus monkeys with and without metabolic syndrome (MetS), among which 45 animals were further assessed by histopathological analysis.

**Results:**

The liver histological features of rhesus monkeys NAFLD were resemble to those of NAFLD patients. There was a close correlation between the histological steatosis grade and the USS (Spearman’s coefficient, 0.705, p < 0.001). The USS sensitivity was 87.5% and the specificity was 94.6% when the cut-off was USS2. In addition, the prevalence of MetS was significantly higher in the USS2–3 group. Multiple risk factors of cardiometabolic disease, including obesity, insulin resistance and dyslipidemia were significantly correlated with the USS.

**Conclusions:**

NAFLD was developed spontaneously among aging in rhesus monkeys (with increased prevalence in the MetS monkeys), which provided an ideal model for NAFLD. The newly developed USS system can be used to evaluate fatty liver in the rhesus monkey. The model as well as the noninvasive assessment methodology will provide a powerful tool for mechanistic studies and preclinical test of novel therapies for NAFLD.

## Background

Non-alcoholic fatty liver disease (NAFLD) is a serious public health problem [1–4]. It has become the most common type of chronic liver diseases in China and western countries [5, 6]. It causes a wide spectrum of histopathological changes, ranging from simple hepatic steatosis to non-alcoholic steatohepatitis (NASH), hepatic cirrhosis and hepatocellular carcinoma [1]. Several long-term follow-up studies demonstrated that higher mortality was observed among patients with NAFLD compared to the general population [7–9], with liver-related diseases, cardiovascular diseases (CVD), and malignancy as the major causes of mortality [7, 10].

Despite intensive clinical and epidemiological studies, the pathogenesis of NAFLD and the interplay between metabolic disease, CVD and other NAFLD related diseases are not fully understood. Moreover, no effective medications have been proved to reverse the liver damage of NAFLD [1]. Adequate animal models of NAFLD are urgently needed for both basic and translational research [11]. As the closest phylogenetic relatives of humans, non-human primates (NHPs) are more similar to humans in terms of lipoprotein profiles, pathogenesis of CVD, response to clinical treatment and genetic makeup [12–15]. Furthermore, the controllability of environmental factors, such as diet, alcohol consumption, and medication, as well as feasibility of tissue sampling and real-time monitoring of disease phenotypes, make NHPs the ideal model for biomedical studies. Rhesus monkeys have been used as the model for alcoholic fatty liver disease [16, 17]. Nagarajan et al. investigated NAFLD in 5 aged bonnet monkeys and 5 aged rhesus monkeys and found that the bonnet monkeys showed a number of biochemical and histopathological characteristics of NAFLD compared to the rhesus monkeys [11]. A couple of research labs including ourselves demonstrated that rhesus monkeys spontaneously developed obesity with aging, metabolic syndrome (MetS), and diabetes [18, 19]. Furthermore, the whole genome of rhesus macaque is available and better annotated now [15, 20–22]. However, naturally occurring NAFLD has not been evaluated and reported in rhesus macaques.

Biopsy is the golden standard for the diagnosis of NAFLD, but its invasiveness and possible complications precluded it as a routine method of assessing NAFLD in humans as well as in animal models. In contrast, noninvasive abdominal ultrasonography is widely used for the screening and diagnosis of fatty liver in the clinic [23–25]. In a prospective study, Saverymuttu et al. [26] have shown that abdominal ultrasonography is a sensitive method for detecting fatty liver. Compared with histological test, ultrasound scanning identifies steatosis with a sensitivity of 94% and a specificity of 84% in human [26]. Furthermore, hepatic ultrasound provides grading of hepatic steatosis based on a sequence of characteristics. Nevertheless, neither features of ultrasonic images nor standards for diagnosis of NAFLD has been reported in rhesus monkeys. In the present study, we aimed to: (1) identify the natural occurrence of NAFLD in a cohort of rhesus monkeys; (2) investigate the histological and ultrasonographic features of NAFLD in rhesus monkeys; (3) establish a hepatic ultrasonographic method for assessing NAFLD in rhesus monkeys; (4) compare the diagnostic accuracy between ultrasonography and histopathology, and verify the ultrasound standard for diagnosing hepatic steatosis in rhesus monkeys; and (5) investigate the relationship between risk factors of MetS and NAFLD in the rhesus monkey model.

## Methods

### Ethics statement

The use and care of the rhesus monkeys were approved and directed by the Animal Care and Use Committee of Peking University and the Association for Assessment and Accreditation of Laboratory Animal Care (Permit Number: IMM-ZhangXQ-1).

### Animals and housing

In this study, we included 86 adult male rhesus monkeys housed in the Laboratory Animal Center of Peking University. The monkeys were housed individually in cages, under a 12-h light–dark cycle at 18–24 °C and 40–70% humidity. The monkeys had free access to water and were fed ad libitum with national standard pellet monkey chow (Beijing HFK Bio-Technology Co., Ltd, China), which contains 7–10% crude fat, 16–20% crude protein, and 55–65% crude carbohydrate.

### Hepatic ultrasonographic imaging

During follow-up observation of the monkeys, hepatic ultrasound images were recorded using a human protocol with modification [25, 27]. In brief, after overnight fasting, the monkeys were anesthetized with ketamine (10 mg/kg, body weight, i.m.) secured in a supine, left lateral position on the testing table with the abdominal hair removed. Hepatic images were acquired by a well-trained technician and recorded with a GE Vivid 7 Dimension ultrasound machine (GE Vingmed Ultrasound, Horten, Norway) using an abdominal transducer (8C). All settings, including over gain and time gain compensation, were kept the same for all monkeys. All sonograms were recorded during smooth breathing and covered different areas of the liver (lobes, edge, portal vein, and hepatic vein), and a contrast image of liver and kidney was recorded. Both the right lobe of the liver and the right kidney were captured side-by-side in one image.

The images of hepatorenal echo contrast were usually obtained under the last right rib. The angle between the probe and the spine was 30–45°. The probe was located in the anterior axillary line in rhesus monkeys, rather than the mid-axillary line in humans [25].

### Parameters for diagnosis of NAFLD

Fatty liver was diagnosed by a clinician specialized in ultrasonography. The ultrasonographic steatosis score (USS) used for diagnosis was based on four findings: hepatorenal echo contrast, bright liver, deep attenuation, and vessel blurring [25, 27]. Hepatorenal echo contrast is the difference of echo between the hepatic and renal parenchyma. Bright liver means brighter and more intense echoes from the hepatic parenchyma. Deep attenuation refers to a reduction in the penetration and amplitude of the ultrasound beam in deeper portions of the liver. And vessel blurring shows narrow lumens and less clear borders of the intrahepatic vessels [23, 25, 27].

### Histological evaluation of fatty liver

During the last few years, there were 22 monkeys euthanized among the cohort because of incurable diseases such as abdominal aortic embolism, myocardial infarction, heart failure, severe diabetes, or severe arthritis. The liver tissue from these monkeys was fixed in 4% paraformaldehyde (PFA), embedded in paraffin, and cut as 5-µm sections followed by staining with hematoxylin-eosin (HE) and Masson’s trichrome. The abdominal ultrasound images were recorded before euthanasia.

In addition, liver biopsy was performed by an experienced surgeon in 23 additional monkeys after abdominal ultrasonography. In brief, after overnight fasting, the monkeys were anesthetized with ketamine (10 mg/kg body weight, i.m.) and was maintained well sedated by inhalation of 2–3% isoflurane during the biopsy. Buprenorphine (0.01 mg/kg, i.m.) was given before and after biopsy for analgesia. Liver samples were fixed in 4% PFA, paraffin-embedded, sectioned, and stained with HE. All sections were evaluated by an experienced pathologist who was blind to the monkeys’ clinical features, and were evaluated semi-quantitatively according to the NAFLD activity score (NAS) [28], which comprised steatosis (0–3): < 5% (0), 5–33% (1), 33–66% (2), and > 66% (3) of steatotic hepatocytes; lobular inflammation (0–3); and hepatocellular ballooning (0–2).

### Blood chemical tests and cytokines/adipokines measurements

The blood samples were taken from a vein before hepatic ultrasonographic imaging. Blood glucose and lipids were measured by a Cobas c 311 analyzer (Roche). Insulin was measured with an insulin measurement kit (Cobas 12017547 122) from Roche using a Cobas e 411 analyzer. TNF-α was measured by Radioimmunoassays with Endothelin radioimmunoassay kit (Beijing North Institute of Biological Technology, China). IL-1b (Invitrogen, USA), IL-2 (Invitrogen, USA), IL-6 (Invitrogen, USA), adiponectin (R&D Systems, USA) and leptin (R&D Systems, USA) were measured by enzyme-linked immunosorbent assays (ELISA) with commercially available ELISA kits.

### Statistical analysis

Continuous variables were expressed as mean ± SE. Categorical variables were described as counts and percentages. Student’s t-test and the χ^2^ test were used in the present study for analysis. Spearman’s correlation coefficients were calculated to evaluate correlations between the USS, histological findings and MetS risk factors. Pearson correlation analysis was performed between the USS and the clinical factors. All statistical tests were two-sided, and the significance level was *p *≤ 0.05. All analyses were performed using SPSS 16.0 (Chicago, IL, USA).

## Results

### Ultrasonic grading of NAFLD and its correlation with histological findings in the rhesus monkeys

**Fig. 1:**
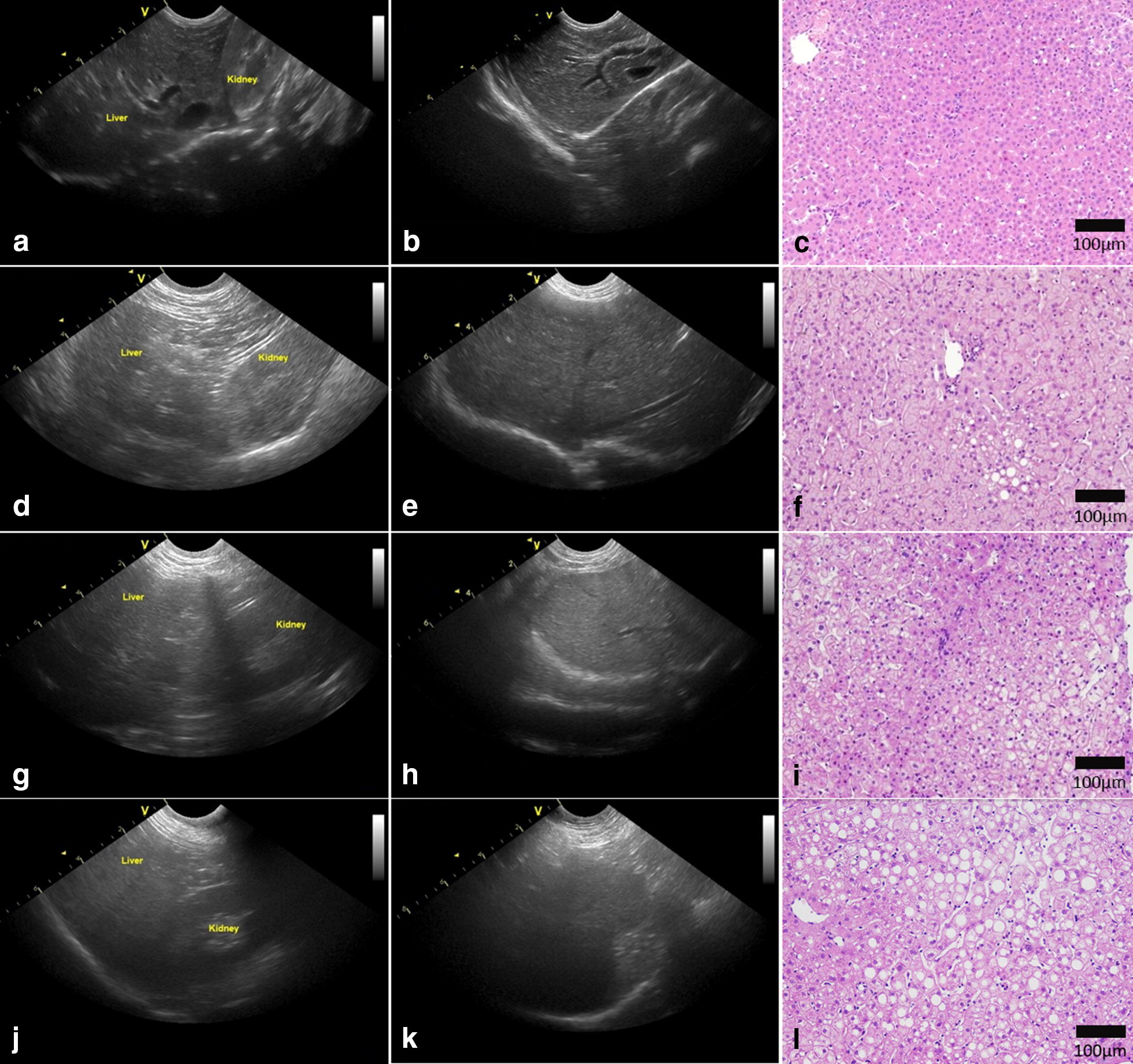
Representative ultrasonographs and histology. Representative ultrasonographs showing different grades of hepatic steatosis in monkeys. **a**, **b** Grade 0, **d**, **e** Grade 1, **g**, **h** Grade 2, and **j**, **k** Grade 3. Representative HE stained sections illustrating a normal liver (**c**), mild (**f**), moderate (**i**), and severe steatosis (**l**)

The ultrasonographic images of the livers were assigned to four grades (0–3) by USS and compared with the histological changes. If the hepatorenal echo contrast was higher in the kidney (Fig. 1a), with no bright liver, no deep attenuation and no vessel blurring (Fig. 1b), it was graded as USS0 (normal), and there is no histological evidence of steatosis (Fig. 1c); if the hepatorenal echo contrast and bright liver were both negative (Fig. 1d) and there was no deep attenuation or vessel blurring (Fig. 1e), it was graded as USS 1 (mild steatosis), and the histology showed that ~ 10% of the cells were steatotic (Fig. 1f); if the hepatorenal echo contrast was higher in liver (Fig. 1g), along with a mild bright liver, negative or mild deep attenuation and negative vessel blurring (Fig. 1h), it was graded as USS2 (moderate steatosis), which with ~ 50% steatotic cells in the liver section (Fig. 1i); and if the hepatorenal echo contrast was much higher in the liver (Fig. 1j), accompanied with a bright liver, attenuation and positive vessel blurring (Fig. 1k), it was graded as USS3 (severe steatosis), and the histological steatosis was almost 80% in the liver section (Fig. 1l).

**Fig. 2:**
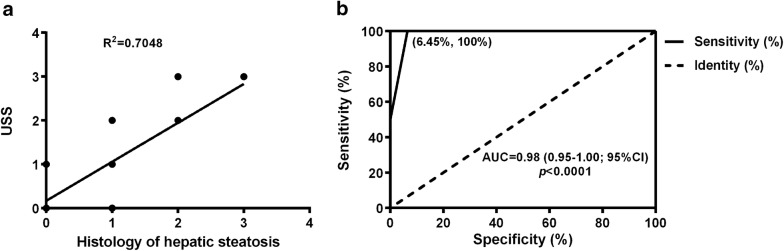
Correlation between USS and histological steatosis. **a** Spearman’s correlation between the USS and histological results of steatosis. **b** Receiver operating characteristic (ROC) curve for the USS to diagnose moderate-to-severe steatosis; cut-off point set at 2 (n = 45; *USS* ultrasonographic steatosis score)

The correlation between the USS and histopathological observation was significant, with a Spearman’s coefficient of 0.705 (*p *< 0.001) (Fig. 2a). Using the USS to diagnose moderate-to-severe steatosis, the area under the receiver operating characteristics curve was 0.98 (Fig. 2b). The percentage of moderate steatosis evaluated by ultrasound matching the histological result was 87.5%. In mild steatosis, the matched USS percentage was 40.0%. The sensitivity and specificity of the different ultrasound scores was provided in Table 1. The sensitivity of USS1 was 43.8%, which was the lowest among the 4 grades, the sensitivity was 87.5% at a cut-off value of USS2 and 100% at USS3. The specificity was 94.6% with a cut-off value of USS2 and 97.44% at USS3. These results suggested that the USS correlates very well with histological changes, especially the sensitivity to moderate and severe hepatic steatosis.

**Table 1:** The accuracy of USS in diagnoses hepatic steatosis

	Sensitivity	95% CI	Specificity	95% CI
USS0	0.60	(0.32–0.84)	0.77	(0.56–0.90)
USS1	0.44	(0.20–0.70)	0.79	(0.60–0.92)
USS2	0.86	(0.47–1.00)	0.95	(0.82–1.00)
USS3	1.00	(0.54–1.00)	0.97	(0.87–1.00)

n = 45; *USS* ultrasonographic steatosis score, *CI* confidence interval

### Typical histological changes in NAFLD monkeys

**Fig. 3:**
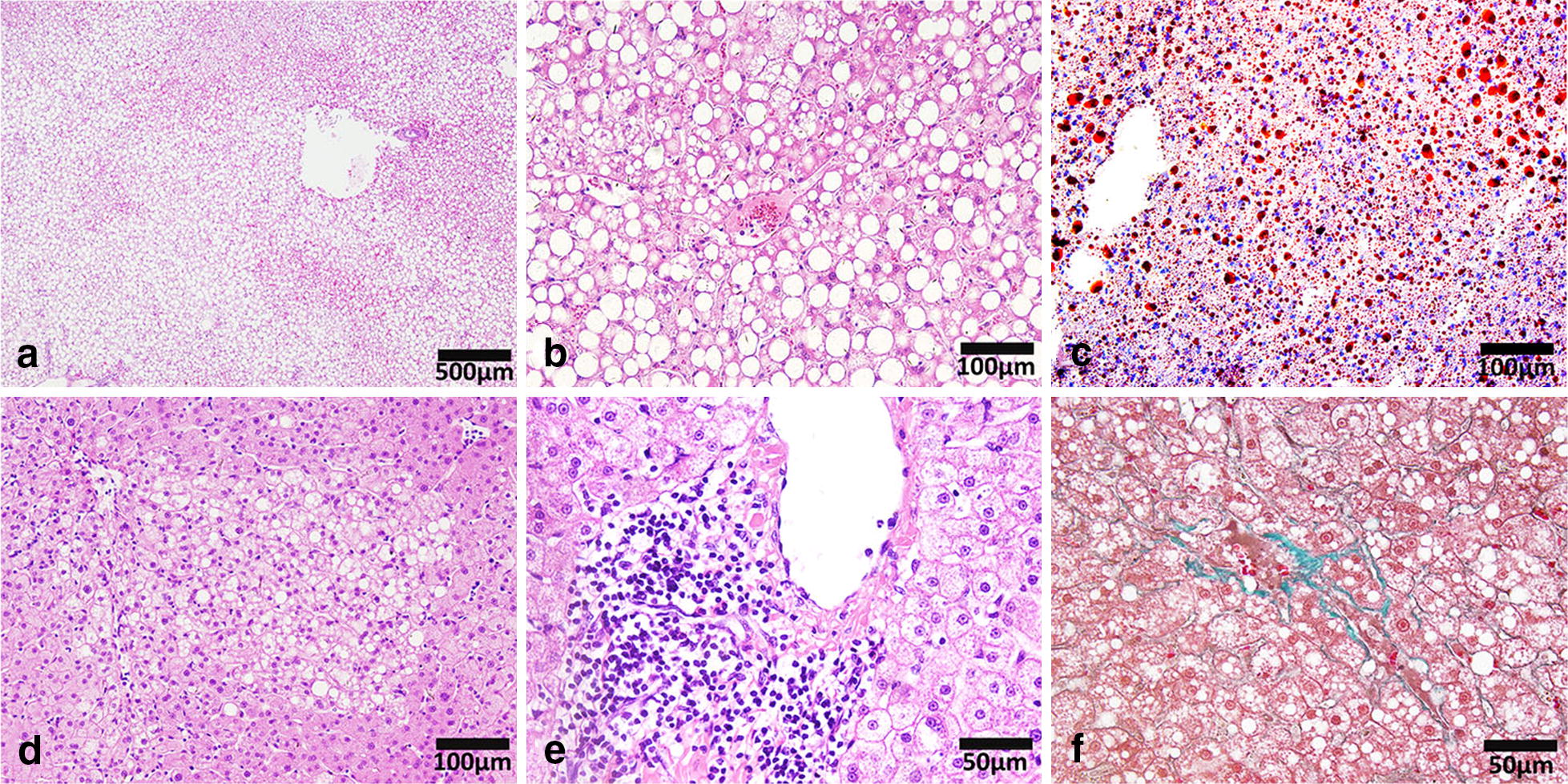
Pathological features of NAFLD in rhesus monkeys. **a**, **b** HE staining showing macrovesicular steatosis in monkey liver sections. **c** Oil Red ‘O’ staining showing the adipose deposition in monkey liver section. **d** HE staining showing microvesicular steatosis in monkey liver section. **e** HE staining showing inflammatory cell infiltration in monkey liver sections. **f** Masson’s trichrome staining showing fibrosis in monkey liver section. (*NAFLD* non-alcoholic fatty liver disease)

The histological characteristics of NAFLD in the monkeys included macrovesicular steatosis (Fig. 3a–c) and microvesicular steatosis (Fig. 3d). Except hepatic steatosis, we also found inflammation (Fig. 3e) and perisinusoidal fibrosis (Fig. 3f). And all these features were similar to the pathological findings in patients with NAFLD and NASH [1]. The specific percentages of different histological changes were shown in Table 2. However, no significant correlation was found between the USS and inflammation or fibrosis.

**Table 2:** The percentage of typical histological changes in NAFLD monkeys

Total	n (%)
	45 (100%)
Steatosis	
< 5%	15 (33.33)
5%–33%	16 (35.56)
33–66%	8 (17.78)
≥ 66%	6 (13.33)
Inflammation	
No foci	18 (40.00)
≤ 2 foci/20 × field	17 (37.78)
2–4 foci/20 × field	9 (20.00)
≥ 4 foci/20 × field	1 (2.22)
Ballooning	
None ballooning cells	39 (86.67)
Few ballooning cells	4 (8.89)
Many ballooning cells	2 (4.44)
Fibrosis	
Perisinusoidal/pericellular fibrosis	7 (15.56)
Periportal fibrosis	4 (8.89)
Bridging fibrosis	0 (0)
Cirrhosis	0 (0)

### Prevalence of hepatic steatosis in the MetS monkeys

**Fig. 4:**
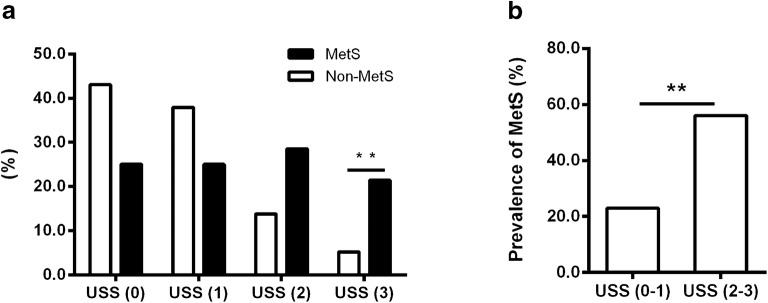
Prevalence of hepatic steatosis in MetS monkeys. **a** Percentages of MetS and non-MetS in normal-to-severe hepatic steatosis groups (non-MetS n = 58; MetS n = 28; *p < 0.05, **p < 0.01). **b** Prevalence of MetS in moderate-to-severe hepatic steatosis, evaluated by the USS (USS0–1, n = 61; USS2–3, n = 25; *p < 0.05, **p < 0.01; *MetS* metabolic syndrome, *USS* ultrasonographic steatosis score)

In our previous study, we have reported that some monkeys spontaneously developed MetS along aging, the MetS monkey was diagnosed when monkey displaying ≥ 3 MetS components. MetS components were: (1) waist circumference (WC) ≥ 40 cm and waist/hip ratio ≥ 0.9, (2) fasting plasma glucose (FPG) ≥ 4.40 mmol/L, (3) triglycerides (TG) ≥ 0.90 mmol/L, (4) high-density lipoprotein cholesterol (HDL-c) ≤ 1.55 mmol/L, (5) blood pressure ≥ 130/80 mmHg [19]. By analyzing the ultrasonic images, we found that the prevalence of hepatic steatosis was significantly higher in the MetS monkeys. Among 28 MetS monkeys, 14 monkeys were moderate-to-severe fatty liver (50.0%). Only 11 out of 58 non-MetS monkeys (19.0%) had NAFLD. The percentages of MetS and non-MetS monkeys in different USS groups were shown in Fig. 4a. 21.4% of the MetS monkeys had severe hepatic steatosis, which was much higher than that in non-MetS monkeys (5.1%). Consistently, the MetS prevalence was significantly higher in the USS2–3 group (56.0%) than in the USS0–1 group (23.0%) (Fig. 4b). The number of MetS risk factors was significantly correlated with the USS (correlation coefficient = 0.305, *p* = 0.004). The incidence of NAFLD and MetS was increased along aging, and monkeys with MetS more susceptible to develop NAFLD spontaneously (Table 3).

**Table 3:** The percentage of NAFLD and MetS in different age groups

Age	< 8	8–14	≥ 15	Total
n	8	8	70	86
Number of USS (2–3)	0	2	23	25
Percentage of NAFLD (%)	0.00	25.00	32.86	29.07
Number of MetS	0	3	25	28
Number of NAFLD in MetS monkeys	0	1	13	14
Prevalence of NAFLD in MetS monkeys (%)	–	33.33	52.00	50.00
Prevalence of NAFLD in Non-MetS monkeys (%)	–	20.00	22.22	18.97

*USS* ultrasonographic steatosis score, *MetS* metabolic syndrome, *NAFLD* non-alcoholic fatty liver disease

### Clinical features of monkeys with different values of the USS

We evaluated the correlation between the USS and clinical features in 86 monkeys (Table 4). Systolic blood pressure, body weight (BW), body mass index (BMI), WC, FPG, homeostasis model assessment of insulin resistance (HOMA-IR), TG, total cholesterol and low-density lipoprotein-cholesterol were significantly higher in the USS2–3 group. HDL-c was clearly lower in the USS2–3 group. There were no significant differences between the two groups in alanine aminotransferase (ALT) and aspartate aminotransferase (AST). BW, BMI, WC, insulin, HOMA-IR, and TG were significantly correlated with the USS, while the Pearson’s correlation coefficients were > 0.35 (Table 5). These results demonstrated that obesity, TG, and insulin resistance (IR) correlated well with the USS.

**Table 4:** Clinical characteristics of monkeys with USS0–1 or USS2–3

Factor	All (n = 86)	USS 2–3 (n = 25)	USS 0–1 (n = 61)	*p*-value
Age (years)	18.1 ± 0.6	17.8 ± 0.5	18.2 ± 0.8	0.76
SBP (mmHg)	127.1 ± 2.3	134.3 ± 4.3	124.1 ± 2.6	0.04
DBP (mmHg)	76.4 ± 1.1	78.9 ± 1.9	75.4 ± 1.4	0.17
BW (kg)	12.8 ± 0.5	16.7 ± 1.0	11.2 ± 0.4	< 0.001
BMI	17.9 ± 0.7	23.5 ± 1.4	15.6 ± 0.5	< 0.001
WC (cm)	45.0 ± 1.5	56.6 ± 3.0	40.2 ± 1.3	< 0.001
FPG (mmol/L)	4.6 ± 0.2	5.2 ± 0.6	4.3 ± 0.1	0.03
HOMA IR	6.9 ± 1.6	14.3 ± 5.1	3.9 ± 0.5	0.003
Insulin (μU/ml)	28.0 ± 3.9	50.1 ± 11.3	19.0 ± 2.1	< 0.001
CRP (mg/dL)	0.3 ± 0.1	0.4 ± 0.1	0.3 ± 0.1	0.26
TG (mmol/L)	0.8 ± 0.1	1.3 ± 0.4	0.6 ± 0.0	0.005
TC (mmol/L)	4.1 ± 0.2	4.9 ± 0.6	3.8 ± 0.2	0.03
HDL-c (mmol/L)	1.9 ± 0.1	1.6 ± 0.1	2.0 ± 0.1	0.01
LDL-c (mmol/L)	2.0 ± 0.2	2.9 ± 0.6	1.7 ± 0.2	0.01
NEFA (mmol/L)	1.1 ± 0.1	1.4 ± 0.3	1.0 ± 0.1	0.10
ALT (U/L)	61.7 ± 4.0	65.1 ± 8.2	60.3 ± 4.5	0.59
AST (U/L)	27.4 ± 0.8	26.5 ± 1.	27.7 ± 0.9	0.52

Data represented as mean ± SE; student *t*-test

*USS* ultrasonographic steatosis score, *SBP* systolic blood pressure, *DBP* diastolic blood pressure, *BW* body weight, *BMI* body mass index, *WC* waist circumference, *FPG* fasting plasma glucose, *HOMA IR* homeostasis model assessment of insulin resistance, *CRP* C-reactive protein, *TG* triglycerides, *TC* total cholesterol, *HDL-c* high-density lipoprotein-cholesterol, *LDL-c* low-density lipoprotein-cholesterol, *NEFA* non-esterified fatty acid, *ALT* alanine aminotransferase, *AST* aspartate aminotransferase

**Table 5:** Correlations between USS and clinical factors

Factor	Pearson r	*p*-value
Age	0.13	0.24
SBP	0.19	0.08
DBP	0.09	0.39
BW	0.61	< 0.001
BMI	0.61	< 0.001
WC	0.61	< 0.001
FPG	0.35	0.001
HOMA IR	0.37	< 0.001
Insulin	0.41	< 0.001
CRP	0.19	0.08
TG	0.38	< 0.001
TC	0.30	0.006
HDL-c	-0.28	0.009
LDL-c	0.31	0.003
NEFA	0.21	0.049
ALT	0.08	0.49
AST	0.004	0.97

*Pearson r* Pearson’s correlation coefficient, *USS* ultrasonographic steatosis score, *SBP* systolic blood pressure, *DBP* diastolic blood pressure, *BW* body weight, *BMI* body mass index, *WC* waist circumference, *FPG* fasting plasma glucose, *HOMA IR* Homeostasis model assessment of insulin resistance, *CRP* C-reactive protein, *TG* triglycerides, *TC* total cholesterol, *HDL-c* high-density lipoprotein-cholesterol, *LDL-c* low-density lipoprotein-cholesterol, *NEFA* non-esterified fatty acid, *ALT* alanine aminotransferase, *AST* aspartate aminotransferase

### Adiponectin was decreased in severe NAFLD monkeys

**Fig. 5:**
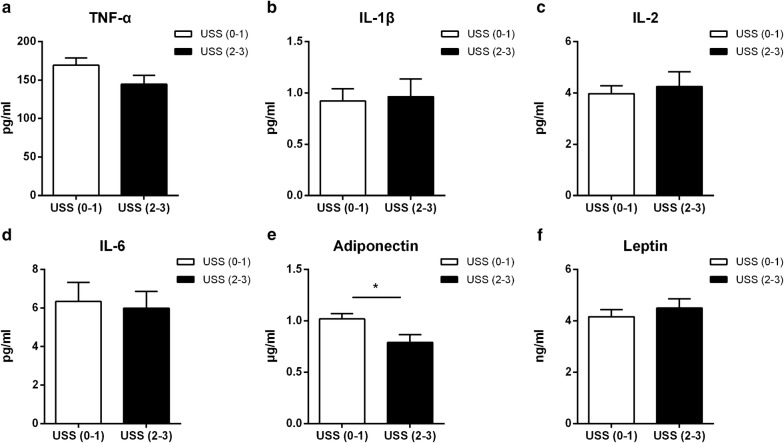
Levels of plasma cytokines and adipokines in different USS groups. **a** TNF-α. **b** IL-1β. **c** IL-2. **d** IL-6. **e** Adiponectin. **f** Leptin. Data are expressed as mean ± SE. USS (0–1), n = 61; USS (2–3), n = 25; *p < 0.05; *USS* ultrasonographic steatosis score

Next, we tested the plasma levels of cytokines and adipokines in NAFLD and non-NAFLD monkeys, and found that TNF-α, IL-1β, IL-2 and IL-6 were no differences between the groups of USS0–1 and USS2–3 (Fig. 5a–d), and no correlation was observed between these cytokines with the USS. Adiponectin was significantly lower in the USS2–3 group (Fig. 5e), and it was negatively associated with the USS (Pearson r = − 0.3113, *p* = 0.0035); while there was no significant difference in leptin between the two groups (Fig. 5F).

## Discussion

In the present study, we found a subpopulation of aged rhesus monkeys that spontaneously developed NAFLD and established an USS standard for evaluating the grade of liver steatosis in rhesus monkeys. The USS matched well with the histopathological changes in the liver, demonstrating that the USS is a useful noninvasive method for quantifying hepatic steatosis in rhesus monkey models with NAFLD. The prevalence of NAFLD was higher in the MetS monkeys, with the USS correlated significantly with the number of MetS risk factors. Obesity, TG, and IR were associated with the grade of liver steatosis. This study provided an excellent animal model for mechanistic and preclinical study of NAFLD.

Animal models, especially NHPs, are ideal animal models for investigating the pathological mechanisms and preclinical studies of NAFLD since alcohol intake could be completely avoided, even in long-term follow-up studies. Ultrasonography is a noninvasive method to evaluate the different processes of NAFLD, and it correlate well with histological changes in clinic [23, 29–32]. However, the ultrasonographic features of the organs are not exactly the same in humans and animals because of the different physiology and anatomy. The evaluation of rhesus monkey NAFLD model with ultrasonography and its correlations with histological of liver steatosis has not been reported. Here, we found that the USS matched well with the histopathological findings from the biopsy and autopsy samples, indicating that the USS is a sensitive and specific method for detecting steatosis in the liver of rhesus monkeys. However, similar to clinical observations, the USS was more accurate when steatosis was more severe. The ultrasonographic features changed when fat filled > 15–20% of the hepatocytes [33]. When the prevalence of hepatic steatosis was > 20%, higher specificity and a greater correlation with the histological findings was evident [34]. In our study, the ultrasonographic results were more consistent with hepatic steatosis when the steatosis was > 33%. These results suggested that noninvasive, easily performed ultrasound and the USS could be used as a screening tool to confirm and evaluate liver steatosis during the development of NAFLD in rhesus monkeys. However, the USS had a poor correlation with inflammation or fibrosis. This is consistent with a previous study in patients, which also found that ultrasonography does not accurately assess the grade of inflammation or NASH [27]. Adiponectin modulates glucose regulation and fatty acid oxidation [35], which was decreased in the USS2–3 group. It was also negatively associated with the USS. This result was consistent with clinical studies [36, 37]. Clinical study suggested that NAFLD is a component in the MetS cluster [38]. A prospective studies over 5 to 6 years of follow-up showed that NAFLD increases the risk of MetS by ~ twofold [39]. The grade of steatosis is much severe when the metabolic disorder is more complicated [40]. Consistent with the clinical studies, we also found the MetS monkeys who had moderate-to-severe hepatic steatosis far more than the non-MetS monkeys. Furthermoer, the prevalence of MetS was significantly higher in the USS2–3 group.

Bellentani et al. [41] found that obese people have a 4.6-fold higher risk of developing liver steatosis. The “two-hit hypothesis” is widely accepted as the pathophysiological mechanism underlying NAFLD [42]. Metabolic disorders are associated with central obesity. IR and compensatory hyperinsulinemia also play a fundamental role in steatosis and even steatohepatitis [38]. This also matched our findings that the USS was positively correlated with BW, BMI, WC, HOMA IR, insulin, and TG in rhesus monkeys. In contrast to the previous report on bonnet monkeys [11], the ALT and AST were not correlated with NAFLD diagnosed by the USS in our study. Nevertheless, these results are in accordant with clinical observations, ALT and AST do not rise in 79% of NAFLD patients [43], and their levels are poor indicators of diagnosis of NAFLD [27].

## Conclusion

Our results demonstrated that the aged rhesus monkeys, especially those with MetS, were more susceptible to develop NAFLD spontaneously, which could be screened using the noninvasive ultrasonic methods.

## Abbreviations

NAFLD: non-alcoholic fatty liver disease
NASH: non-alcoholic steatohepatitis
CVD: cardiovascular diseases
NHP: non-human primate
MetS: metabolic syndrome
USS: ultrasonographic steatosis score
PFA: paraformaldehyde
HE: hematoxylin–eosin
NAS: NAFLD activity score
ELISA: enzyme-linked immunosorbent assays
WC: waist circumference
FPG: fasting plasma glucose
TG: triglycerides
HDL-c: high-density lipoprotein cholesterol
BW: body weight
BMI: body mass index
HOMA-IR: homeostasis model assessment of insulin resistance
ALT: alanine aminotransferase
AST: aspartate aminotransferase
IR: insulin resistance
